# Probiotics alleviate oral microbiota disruptions induced by *Helicobacter pylori* eradication with vonoprazan-amoxicillin dual therapy: a randomized double-blind placebo-controlled trial. (An exploratory study)

**DOI:** 10.3389/fcimb.2026.1740968

**Published:** 2026-05-13

**Authors:** Wenjuan Wei, Weiwei Chen, Ruolin Peng, Xiujuan Wang, Zhibin Wang, Zhenyu Zhang

**Affiliations:** 1 Department of Gastroenterology, Nanjing First Hospital, Nanjing Medical University, Nanjing, Jiangsu, China; 2 Department of Gastroenterology, Sihong First Hospital, Suqian, Jiangsu, China

**Keywords:** eradication, *Helicobacter pylori*, oral microbiota, probiotics, vonoprazan-amoxicillin dual therapy

## Abstract

**Background:**

*Helicobacter pylori* (*H. pylori*) infection affects approximately half of the global population and has been linked to oral microbiota dysbiosis, while eradication therapies may further disrupt microbial balance. This exploratory study investigated the effects of *H. pylori* infection, 14-day vonoprazan-amoxicillin (VA) dual therapy, and adjunctive probiotics (live combined Bacillus subtilis-Enterococcus faecium enteric-coated capsules) on oral microbiota.

**Methods:**

A total of 78 *H. pylori*-positive patients were randomized to the PT group (VA + probiotics) or VA group (VA + placebo), with 20 *H. pylori*-negative volunteers as controls. Tongue coating samples were collected at baseline (W0), post-treatment (W2), and follow-up (W6) for 16S rRNA sequencing.

**Results:**

Results showed that *H. pylori* infection significantly reduced oral microbial diversity (Chao1 and Shannon indices) and altered community structure (β-diversity) compared to controls, with decreased Hemophilus and Prevotella and impaired carbohydrate metabolic pathways. The 14-day VA regimen transiently reduced α-diversity (Chao1 index) and perturbed β-diversity, with increased Actinobacteria and decreased Bacteroidetes at W2, followed by partial recovery by W6. Notably, the PT group exhibited smaller diversity fluctuations, stable β-diversity, and partially mitigate phylum-level perturbations compared to the VA group, alongside suppressed pathogenic genera (e.g.,Fusobacterium, Mycoplasma) and enhanced carbohydrate metabolism pathways. However, multiple diversity metrics and compositional profiles in both intervention groups remained significantly different from the H. pylori-negative control group at post-treatment (W2) and follow-up (W6) time points.

**Conclusion:**

In conclusion, H. pylori infection disrupts oral microbiota, and 14-day VA therapy induces transient microbial perturbations. Adjunctive probiotics partially mitigate these disruptions, supporting oral microecological stability during eradication therapy, but do not achieve full restoration of microbiota balance to the level of uninfected individuals in the short term. These effects are partial, as complete restoration to uninfected microbiota levels was not achieved within the study follow-up period.

Chinese Clinical Trial Registry (ChiCTR2400082446).

## 1 Introduction

As the gateway to the digestive system, the oral cavity hosts a diverse microbial community that includes bacteria, fungi, and viruses (Mark Welch et al., 2019). The oral microecology is closely associated with the health status of the human body. When its homeostasis is disrupted, the number of pathogenic bacteria in the oral cavity increases, thereby inducing oral, gastrointestinal, and other related diseases (Hakansson et al., 2018).

*H. pylori*, a gastric pathogenic bacterium and primary gastric cancer risk factor, infects ~50% of the global population (Hu et al., 2022). Eradicating *H. pylori* may modify gastric/intestinal microbiota due to proton-pump inhibitors (PPIs) and antibiotics in therapies (He et al., 2019). Multiple reports show *H. pylori* infection alters oral microbiota, with diversity/composition differences between infected and non-infected individuals; eradication therapy also changes oral microbiota (Chua et al., 2019; Peng et al., 2023). Different *H. pylori* eradication protocols disrupt microbiota to varying extents: Bismuth-containing quadruple therapy (BQT) perturbs oral microbiota community composition and structure (Ji et al., 2020). An investigation demonstrates that 7-day triple therapy for *H. pylori* eradication induces an immediate diminishment in oral flora diversity (Jakobsson et al., 2010).

Vonoprazan-amoxicillin (VA) dual therapy is a first-line, clinically effective H. pylori treatment, causing less gut microbial disruption than triple/quadruple regimens (Horii et al., 2021). A study reported that the VA dual therapy regimen exerted regulatory effects on the diversity, structural configuration, and functional profiles of the oral microbial community (Peng et al., 2023). Nevertheless, a separate investigation demonstrated that the VA dual therapy exerted minimal impact on the oral microbial flora (Hu et al., 2022). In fact, The 14-day VA protocol reduces one antibiotic vs. standard regimens but increases amoxicillin dosage; vonoprazan also has stronger, longer acid suppression than conventional proton pump inhibitors. Hence, further studies are needed to assess its oral microbiota impact.

Beneficial bacteria, as a class of live microorganisms, can exert positive effects on human health. These microorganisms are capable of modulating the microbiota, strengthening barrier function, regulating immunity and metabolism, and are critical for preserving microbial ecological balance. A study indicated that the probiotic quadruple regimen mitigated the dysbiosis of oral microbiota induced by BQT (He et al., 2022). The live combined Bacillus subtilis and Enterococcus faecium enteric-coated capsules represents one of the most frequently utilized probiotic formulations in recent years (Payne et al., 2024). The advantageous impacts of this formulation on human microbiota and overall health have been substantiated through extensive research (Wu et al., 2019). While existing investigations into above probiotics primarily center on intestinal effects, compelling evidence indicates their beneficial actions extend to microbial communities in other anatomical sites such as the nasal cavity and gastric environment (Zeng et al., 2016; Piewngam et al., 2023). Thus, exploring if Bacillus subtilis-Enterococcus faecium enteric-coated capsules mitigate oral dysbiosis from *H. pylori* eradication therapy is worthwhile.

This prospective exploratory pilot study investigated the effects of *H. pylori* infection, 14-day VA eradication therapy, and adjunctive Bacillus subtilis-Enterococcus faecium probiotics on oral microbiota dysbiosis. Based on prior literature, 30 participants per group were required to achieve 80% power (α=0.05) to detect a 15% Chao1 index difference. The final sample of 31 PT-group and 33 VA-group patients satisfied the statistical power requirement for exploratory analysis. Notably, our study differs from previous work in three key aspects: (1) we specifically evaluate the 14-day VA dual therapy regimen with higher amoxicillin dosage, which has become the first-line eradication protocol in many regions; (2) we provide detailed longitudinal analysis of microbial recovery trajectories at both phylum and genus levels, including functional pathway alterations; (3) we directly compare the effects against a well-characterized H. pylori-negative control group to contextualize the magnitude of microbial perturbations and recovery.

## 2 Materials and methods

### 2.1 Study design and ethical considerations

A single-center, prospective small-sample exploratory investigation was performed, which included 78 *H. pylori*-positive patients clinically diagnosed at the Gastroenterology Outpatient Department of Nanjing First Hospital from April 1, 2024 to August 1, 2024 as research participants. Besides, 20 *H. pylori*-negative healthy volunteers were chosen from the hospital’s health management center to act as the negative control group for the analysis of tongue coating microbiota. The study protocol was registered at Chinese Clinical Trial Registry (ChiCTR2400082446). This study was approved by the Ethics Committee of Nanjing First Hospital (Approval Number: KY20240123-20).

The negative control group was recruited from individuals undergoing routine health examinations who matched the H. pylori-infected patients in geographic location, recruitment period, and general inclusion criteria (age 18–65 years, no systemic comorbidities, no recent antibiotic use). While the control group showed a trend toward younger age (p=0.072, **Table 1**), this difference was not statistically significant, and all participants were within the same adult age range with no known oral or systemic conditions that would independently affect oral microbiota. Sensitivity analysis adjusting for age, BMI, and gender was performed using permutational multivariate analysis of variance (PERMANOVA), which confirmed that H. pylori infection status remained the primary predictor of oral microbiota composition (R²=0.082, p=0.002) after controlling for these demographic variables.

**Table 1:** General characteristics of patients in different groups included in tongue coating microbiota detection.

General characteristics	PT group (n=31)	VA group (n=33)	Negative control group (n=20)	P value
Age	43.68 ± 12.86	39.33 ± 10.91	36.1 ± 10.74	0.072
Gender (Male/Female)	9/22	12/21	7/13	0.811
BMI (kg/m²)	23.24 ± 3.00	23.16 ± 3.25	22.12 ± 3.08	0.404
Smoking history	2/31(6.5%)	5/33(15.2%)	0/20(0.0%)	0.154
Alcohol consumption history	1/31(3.2%)	3/33(9.1%)	0/20(0.0%)	0.443

### 2.2 Study population

Inclusion Criteria: 1) Aged 18–65 years; 2) Confirmed *H. pylori*-positive; 3) Never received *H. pylori* eradication therapy or prior failure with no treatment in the past 6 months; 4) Voluntarily consented and signed informed consent. Exclusion Criteria: 1) Allergic to study drugs (e.g., penicillin); 2) Chronic gastritis with peptic ulcer; 3) Eradication therapy within 6 months; 4) Antibiotics/bismuth use within 4 weeks, or H_2_ receptor antagonists/PPIs within 2 weeks before enrollment; 5) Current use of corticosteroids, NSAIDs, or anticoagulants; 6) History of esophageal/gastric surgery; 7) Pregnant/lactating women; 8) Severe comorbidities (liver, cardiovascular, pulmonary, renal disease); 9) Alcohol abuse.

### 2.3 Randomization and double-blinding

In this clinical trial, the biostatistical team designed the random allocation sequence using SAS 9.4 software with a block randomization method (block size 6). Eligible participants were randomly assigned at a 1:1 ratio to two groups: the PT group got the vonoprazan-amoxicillin regimen with probiotics, and the VA group got the same regimen with a placebo. All medications were given according to random codes, keeping both participants and investigators blinded during the 14-day treatment.

### 2.4 Intervention and control

PT group: Oral vonoprazan fumarate (20 mg twice daily), amoxicillin (1000 mg thrice daily), and live Bacillus subtilis-Enterococcus faecium enteric-coated capsules (500 mg thrice daily). 2) VA group: Identical vonoprazan-amoxicillin regimen plus starch-based placebo (500 mg thrice daily), matching probiotics in weight, appearance, and taste but lacking active ingredients. Both groups received 14-day *H. pylori* eradication therapy. Probiotics/placebo (supplied by Beijing Hanmi Co., Ltd.) were administered ≥2 hours apart from amoxicillin.

The probiotic capsules (Beijing Hanmi Pharmaceutical Co., Ltd., China) contain 1.5×10^7^CFU of *Bacillus subtilis*(strain CMCC(B) 63501) and 1.5×10 CFU of *Enterococcus faecium* (strain CMCC(B) 63502) per capsule. Viability testing was conducted prior to the study, confirming ≥ 90% survival rate under the recommended storage conditions (2-8 °C) throughout the trial period.

### 2.5 Sample collection

Tongue coating samples were collected from *H. pylori*-positive and -negative patients at W0 (pre-intervention), W2 (post-eradication) and W6 (cure confirmation) following standard sampling protocols, and stored at −80 °C for analysis. W6 was selected as the follow-up time point in accordance with clinical guidelines for *H. pylori* eradication efficacy assessment (Malfertheiner et al., 2022). This time point allowed us to correlate microbial recovery patterns with the clinically confirmed eradication status of patients. While we acknowledge that oral microbiota may require longer periods to achieve complete stability following antibiotic perturbation, this exploratory study prioritized alignment with clinical endpoint assessment to generate findings with direct translational relevance for *H. pylori* treatment protocols.

### 2.6 DNA extraction and sequencing

Total genomic DNA was extracted via CTAB. Concentration/purity were measured using a NanoDrop 2000 spectrophotometer, and integrity assessed by 1% agarose gel electrophoresis (5 V/cm, 20 min). The V3-V4 hypervariable region of the 16S rRNA gene was amplified with specific primers (338F: ACTCCTACGGGAGGCAGCAG; 806R: GGACTACHVGGGTWTCTAAT). The 30 μL PCR system contained 15 μL Phusion^®^ High-Fidelity PCR Master Mix, 3 μL each primer, ~10 ng template DNA, and ddH_2_O. Thermal cycling conditions: initial denaturation at 95°C for 3min; 30 cycles of 95°C (30 s), 50°C (30 s), 72°C (45 s); final extension at 72°C for 10min. Triplicate PCR products per sample were pooled, visualized by 2% agarose gel electrophoresis, and purified using the GeneJET Gel Extraction Kit. Libraries were constructed with the TruSeq DNA PCR-Free Library Preparation Kit and sequenced on the Illumina NovaSeq 6000 platform (PE250 paired-end strategy).

### 2.7 Sequencing analysis

Raw sequencing data were demultiplexed using QIIME1 (v1.8.0), followed by quality control and sequence merging via the PEAR (v0.9.6) and VSEARCH (v2.7.1) tools. Subsequently, the merged sequences were denoised using the UNOISE3 method to generate amplicon sequence variants (ASVs). Finally, all sequences were taxonomically annotated by aligning against the SILVA 138 database using the RDP Classifier algorithm. The raw reads were deposited into the NCBI Sequence Read Archive (SRA) database (Accession Number: PRJNA1450734).

### 2.8 Bioinformatics and statistical analysis

Based on ASV data, α-diversity indices (Chao1 richness index, Shannon diversity index) were calculated using QIIME 1 (v1.8.0). Principal coordinate analysis (PCoA) and partial least squares discriminant analysis (PLS-DA) based on Bray-Curtis distances were performed using R (v3.6.0) to assess community structure similarity across samples. Linear discriminant analysis effect size (LEfSe) was used to identify differential biomarker taxa between *H. pylori*-positive and -negative groups, with an LDA score > 3.0 set as the significance threshold.

Differences in bacterial genera across baseline, post-eradication and follow-up time points were tested via t-tests, and significantly differential genera were visualized using heatmaps and Venn diagrams. All multiple comparisons across bacterial taxa were adjusted for false discovery rate (FDR) using the Benjamini-Hochberg procedure, with adjusted p < 0.05 considered statistically significant. Microbial functions were predicted against the KEGG database using PICRUST2. These functional predictions are based on 16S rRNA gene sequencing data and represent inferred metabolic potential rather than direct measurements of functional activity. All multiple comparisons across bacterial taxa were adjusted for false discovery rate (FDR) using the Benjamini-Hochberg procedure, with adjusted p < 0.05 considered statistically significant. For α-diversity comparisons, non-parametric Kruskal-Wallis tests were used as a robustness check alongside t-tests, and consistent results were observed across both methods. ASV tables were rarefied to equal sequencing depth prior to diversity calculations to account for differences in sequencing coverage.

## 3 Results

### 3.1 Features of subjects

**Figure 1:**
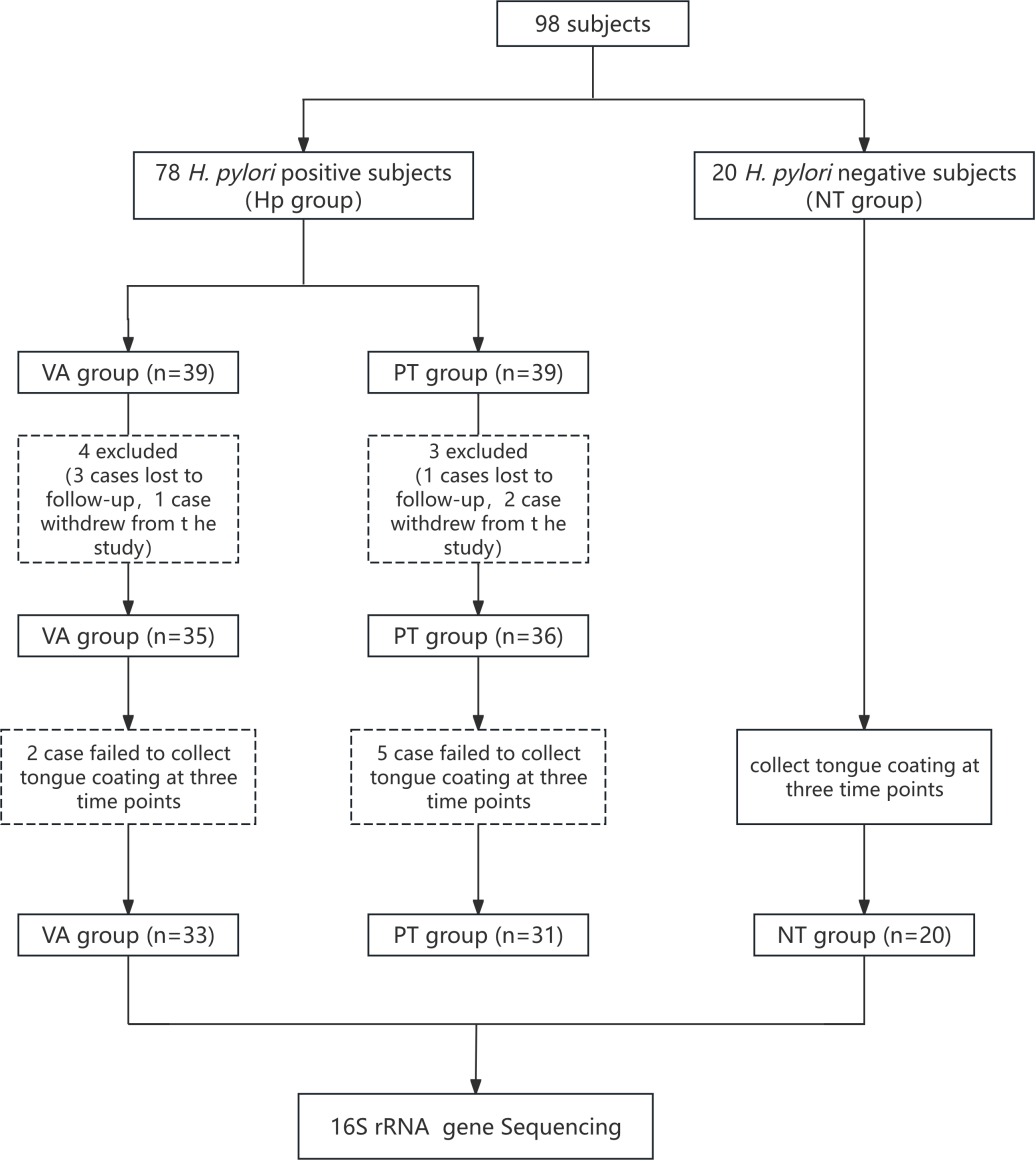
Flow chart of the study. VA group: vonoprazan-amoxicillin regimen plus placebo; PT group: vonoprazan-amoxicillin regimen plus probiotics.

This study enrolled a total of 98 subjects, among whom 78 were diagnosed with *H. pylori* infection via carbon-13 urea breath test at Nanjing First Hospital. They were randomly assigned to the probiotic group or dual therapy group at a 1:1 ratio. Additionally, 20 healthy individuals with negative carbon-13 urea breath test results were recruited from the hospital’s health management center as the negative control group for tongue coating microbiota analysis. In the PT group: 1 subject was lost to follow-up, 2 withdrew early due to adverse reactions, and 36 completed medication, follow-up, and re-examination; In the VA group: 3 subjects were lost to follow-up, 1 withdrew early due to adverse reactions, and 35 completed medication, follow-up, and re-examination. Ultimately, 64 patients successfully eradicated *H. pylori* and had their tongue coating collected three times, including 31 subjects in the PT group and 33 subjects in the VA group (**Figure 1**). There were no significant differences between the groups in demographic characteristics (e.g., age, gender, BMI, smoking, and alcohol consumption history) among subjects included in the ITT analysis or tongue coating microbiota testing (**Tables 1**, **2**).

**Table 2:** Comparison of general characteristics between the two groups.

General characteristics	PT group (n=39)	VA group (n=39)	P value
Age	43.56 ± 12.81	40.23 ± 10.99	0.221
Gender (Male/Female)	10/29	14/25	0.326
BMI (kg/m²)	23.34 ± 2.73	23.32 ± 3.14	0.966
Smoking history	4/39 (10.3%)	6/39 (15.4%)	0.735
Alcohol consumption history	2//39 (5.1%)	4/39 (10.3%)	0.671

### 3.2 Eradication rate and adverse reactions

All enrolled patients were included in the ITT analysis; those with complete medication records were included in the mITT analysis; and patients who took >80% of the full regimen dose and completed follow-up were included in the PP analysis. The eradication rates in the ITT, mITT, and PP analyses were 87.2% vs. 84.6% (p=0.745), 89.5% vs. 91.7% (p=1.000), and 94.4% vs. 94.3% (p=1.000) for the PT and VA groups, respectively. No significant differences were observed between the groups across the analyses. (**Table 3**) 10 patients in the PT group reported adverse events post-treatment. In the VA group, 13 patients reported adverse events after medication. (**Table 4**) Except for three patients who developed rash or eyelid edema (suspected allergic reactions) and were advised to discontinue medication initially by the physician, all other patients had mild symptoms and successfully completed the eradication regimen. Additionally, the incidence of adverse events was higher in the VA group than in the PT group (36.1% vs. 26.3%), but this difference was not statistically significant (p=0.363).

**Table 3:** Comparison of eradication rates between PT group and VA group.

Analysis method	PTgroup	VA group	P value
ITT analysis	34/39 (87.2%)	33/39 (84.6%)	0.745
mITT analysis	34/38 (89.5%)	33/36 (91.7%)	1.000
PP analysis	34/36 (94.4%)	33/35 (94.3%)	1.000

**Table 4:** Comparison of adverse reactions between PT group and VA group.

Adverse reactions	PT group (%)	VA group (%)	P value
Nausea	2 (5.3)	3 (8.3)	0.950
Acid regurgitation	1 (2.6)	0 (0.0)	1.000
Abdominal pain	2 (5.3)	2 (5.6)	1.000
Diarrhea	0 (0.0)	2 (5.6)	0.450
Abdominal distension	1 (2.6)	3 (8.3)	0.569
Vomiting	0 (0.0)	1 (2.8)	0.978
Rash	2 (5.3)	0 (0.0)	0.498
Dizziness	2 (5.3)	0 (0.0)	0.498
Anorexia	0 (0.0)	1 (2.8)	0.978
Left upper eyelid edema	0 (0.0)	1 (2.8)	0.978
Total	10 (26.3)	13 (36.1)	0.363

### 3.3 *H. pylori* infection and oral microbiota

#### 3.3.1 Diversity analysis

**Figure 2:**
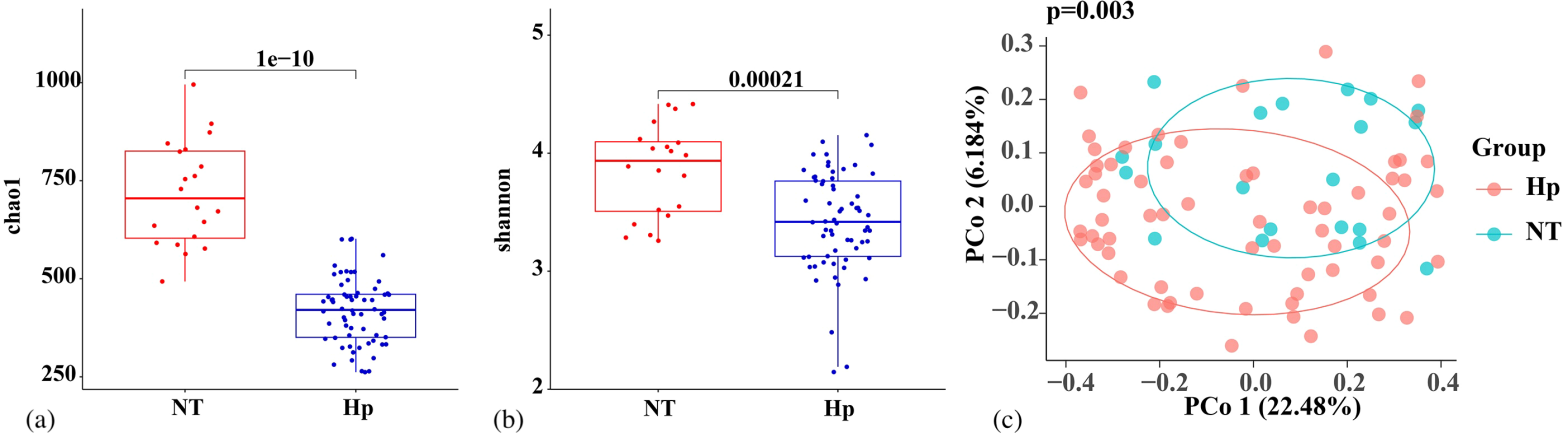
Comparison of tongue coating microbiota diversity between *H. pylori*-negative controls and *H. pylori*-infected patients. Alpha diversity was evaluated using **(a)** Chao1 index and **(b)** Shannon index to compare species richness and evenness between groups. **(c)** Beta diversity was analyzed via principal coordinate analysis (PCoA) based on Bray-Curtis distances to assess differences in community structure. Hp group: *H. pylori*-positive patients (pre-treatment tongue coating samples from both PT and VA groups); NT group: *H. pylori*-negative healthy controls.

Tongue coating microbiota from 20 *H. pylori*-negative subjects (NT group, derived from *H. pylori*-negative healthy individuals undergoing physical examinations at the health management center of Nanjing First Hospital.) and 64 *H. pylori*-infected patients (Hp group) before treatment were subjected to 16S rRNA sequencing. The α-diversity of tongue coating microbiota was evaluated using the Chao1 index (**Figure 2A**) and Shannon index (**Figure 2B**). Results demonstrated that both the Chao1 index and Shannon index in the NT group were significantly higher than those in the Hp group (p < 0.001), suggesting that *H. pylori* infection significantly reduced the species diversity and richness of the oral microbiota in patients. PCoA analysis based on Bray-Curtis distance (**Figure 2C**) revealed a significant difference in β-diversity between the two groups (p = 0.003), indicating distinct structural differences in tongue coating microbiota between *H. pylori*-negative subjects and *H. pylori*-infected individuals. Sensitivity analysis adjusting for age, BMI, and gender was performed using permutational multivariate analysis of variance (PERMANOVA), which confirmed that *H. pylori* infection status remained the primary predictor of oral microbiota composition (R²=0.082, p=0.002) after controlling for these demographic variables.

#### 3.3.2 Bacterial abundance and composition analysis

**Figure 3:**
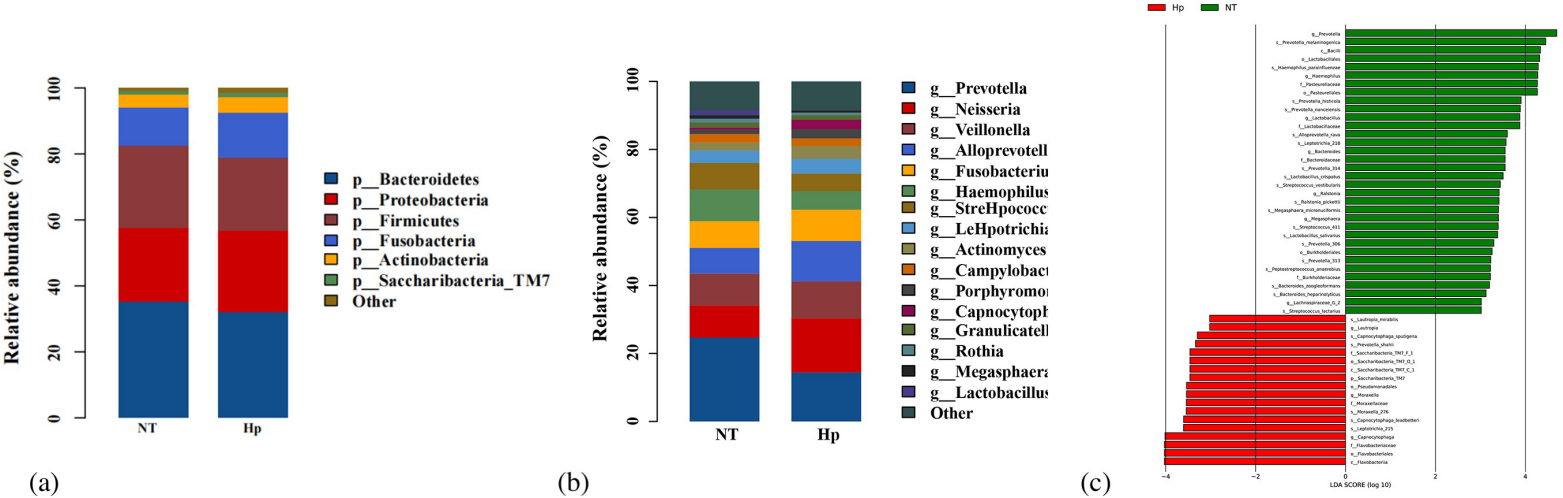
Comparison of tongue coating microbiota composition and structure between *H. pylori*-negative controls and *H. pylori*-infected patients. **(a)** Phylum-level composition of tongue coating microbiota in the two groups. **(b)** Genus-level composition of tongue coating bacterial communities in the two groups. **(c)** LEfSe identifying biomarker taxa distinguishing the two groups. Hp group: *H. pylori*-positive patients; NT group: *H. pylori*-negative healthy controls.

The top five dominant bacterial phyla in both the NT group and Hp group were identical, including Actinobacteria, Fusobacteria, Proteobacteria, Firmicutes, and Bacteroidetes, with no significant differences observed in their relative abundances between the two groups. (**Figure 3A**) The top five genera in the NT and Hp groups are presented in **Figure 3B**. Among these, Hemophilus and Prevotella abundances were significantly higher in NT group. LEfSe analysis identified NT group biomarker phyla/genera: Prevotella, Hemophilus, Lactobacillus, Bacteroides, Ralstonia, Megasphaera, Lachnospiraceae_G_2; Hp group biomarker phyla/genera: Lautropia, Saccharibacteria (TM7), Moraxella, Capnocytophaga. In conclusion, oral microbiota composition differs between *H. pylori*-infected patients and healthy individuals. (**Figure 3C**) In conclusion, oral microbiota composition differs between *H. pylori*-infected patients and healthy individuals.

#### 3.3.3 Characterization of carbohydrate metabolic capacity in oral microbiota

**Figure 4:**
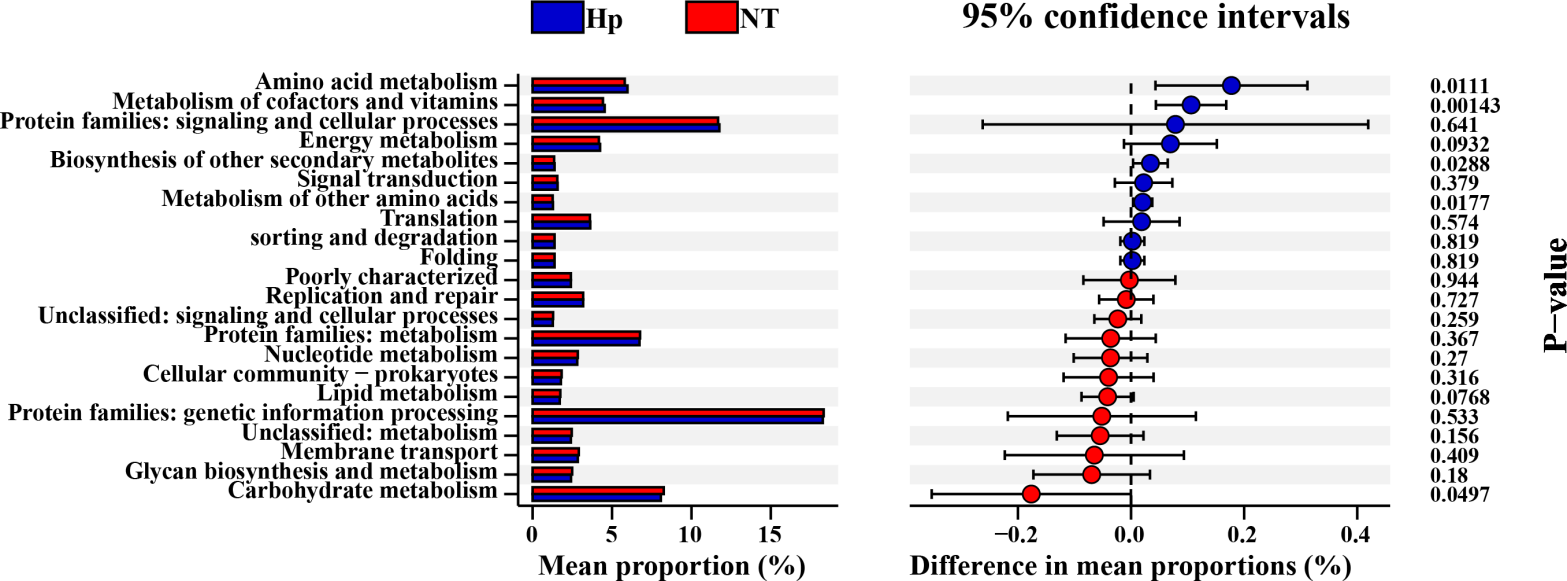
KEGG pathway enrichment analysis of tongue coating microbiota in *H. pylori*-negative controls versus *H. pylori*-positive patients. Hp group, *H. pylori*-positive patients; NT group, *H. pylori*-negative healthy controls.

KEGG pathway enrichment analysis was performed using the sequencing data of tongue coating microbiota from the two groups, and comparison revealed five pathways with significantly differential expression between the two groups. Among these, the expression level of the Carbohydrate metabolism pathway was higher in the NT group than in the Hp group, whereas the expression levels of the Metabolism of other amino acids, Metabolism of cofactors and vitamins, Biosynthesis of other secondary metabolites, and Amino acid metabolism pathways were lower in the NT group than in the Hp group (**Figure 4**). These functional predictions are based on 16S rRNA gene sequencing data and represent inferred metabolic potential rather than direct measurements of functional activity.

### 3.4 *H. pylori* eradication and oral microbiota

#### 3.4.1 The 14-day VA regimen reduced oral microbial diversity

**Figure 5:**
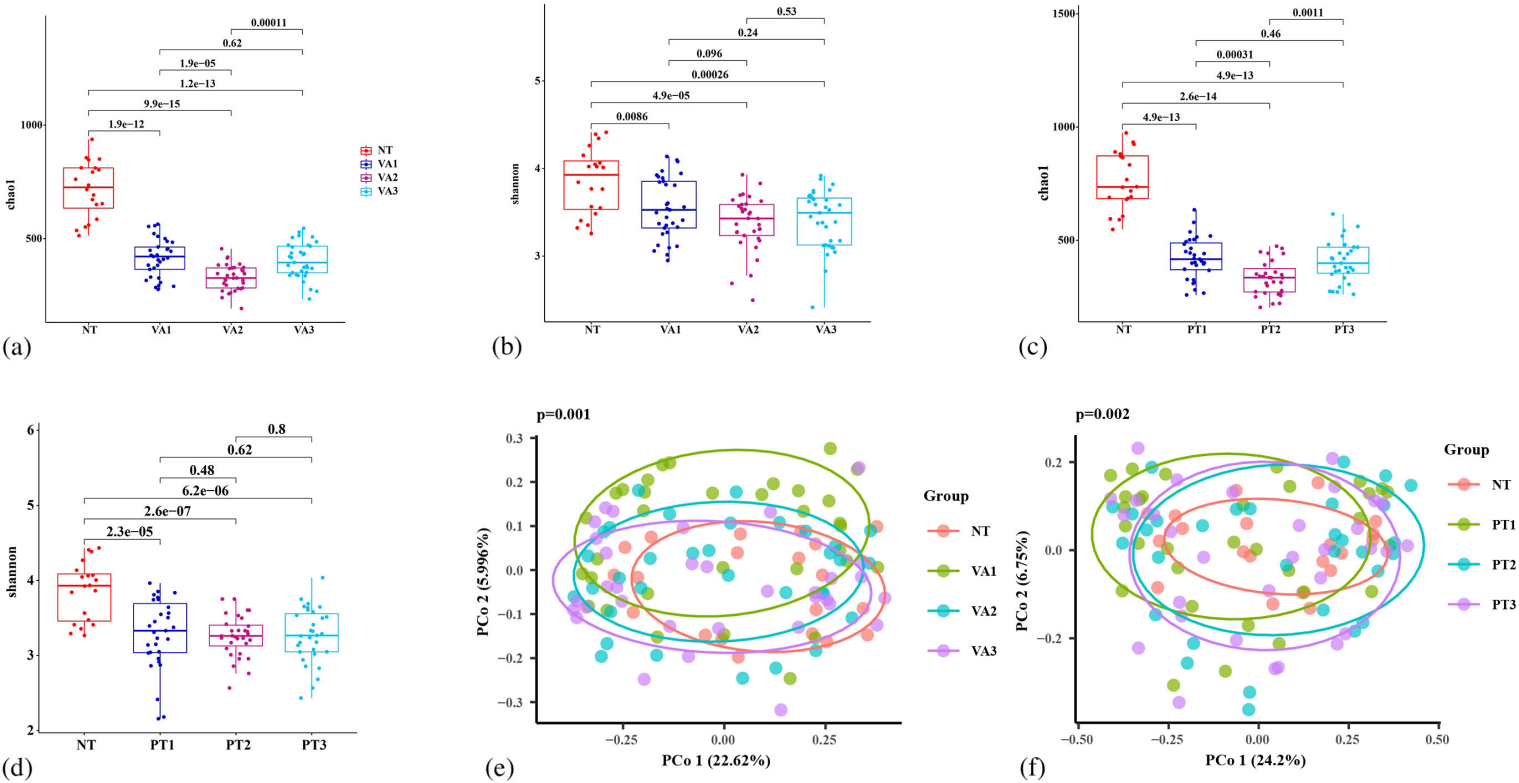
Comparison of tongue coating microbiota diversity between VA and PT groups. **(a)** Chao1 index in VA group before and after treatment; **(b)** Shannon index in VA group before and after treatment; **(c)** Chao1 index in PT group before and after treatment; **(d)** Shannon index in PT group before and after treatment; **(e)** PCoA analysis in VA group before and after treatment; **(f)** PCoA analysis in PT group before and after treatment. VA group: vonoprazan-amoxicillin + placebo. VA1 (baseline, week 0, before eradication); VA2 (post-treatment, week 2, end of therapy); VA3 (follow-up, week 6, re-examination). PT group: vonoprazan-amoxicillin + probiotics. PT1 (baseline, week 0, before eradication); PT2 (post-treatment, week 2, end of therapy); PT3 (follow-up, week 6, re-examination).

During this study, tongue coating microbiota samples were successfully collected from 33 patients in the VA group at three time points: W0, W2, and W6, and 16S rRNA sequencing was performed. α-diversity of the tongue coating microbiota was assessed using the Chao1 index (**Figure 5A**) and Shannon index (**Figure 5B**) across baseline, post-treatment, and follow-up time points. α-diversity: Chao1 index significantly decreased at W2 vs W0 (p<0.001), recovered at W6 (p=0.62); Shannon index showed no significant differences across time points. β-diversity (PCoA, Bray-Curtis distance): microbial community structure changed significantly at W2 vs W0 (p=0.031), recovered at W6 (p=0.093). (**Figure 5E**) These results collectively suggest that the 14-day VA therapy reduced oral microbiota diversity and altered the overall microbial community structure.

#### 3.4.2 Probiotics can partially mitigate the disruption of oral microbial diversity caused by the 14-day VA regimen

Tongue coating microbiota samples from 31 PT group patients were collected at W0, W2 and W6 for 16S rRNA sequencing.α-diversity was assessed via Chao1 (**Figure 5C**) and Shannon indices (**Figure 5D**): the Chao1 index decreased significantly at W2 (p < 0.001) and recovered to baseline at W6 (p = 0.46), while no significant difference was observed in the Shannon index across the three time points, with a smaller fluctuation magnitude than that in the VA group. β-diversity analysis via Bray-Curtis PCoA showed no significant changes in microbial community structure in the PT group at W2 (p = 0.055) or W6 (p = 0.229) compared with W0 (**Figure 5F**). These results indicate that probiotics partially mitigate the disruption of oral microbial diversity and community structure induced by the 14-day VA regimen.

However, compared with *H. pylori*-negative controls, both α-diversity (Chao1, Shannon) and β-diversity profiles of the PT group remained significantly different at W2 and W6, suggesting probiotic supplementation fails to fully restore oral microbiota to pre-infection levels within the study follow-up period.

#### 3.4.3 Effects of different treatment regimens on the composition of core oral microbiota

##### 3.4.3.1 The VA regimen altered the relative abundance of core oral bacterial phyla

**Figure 6:**
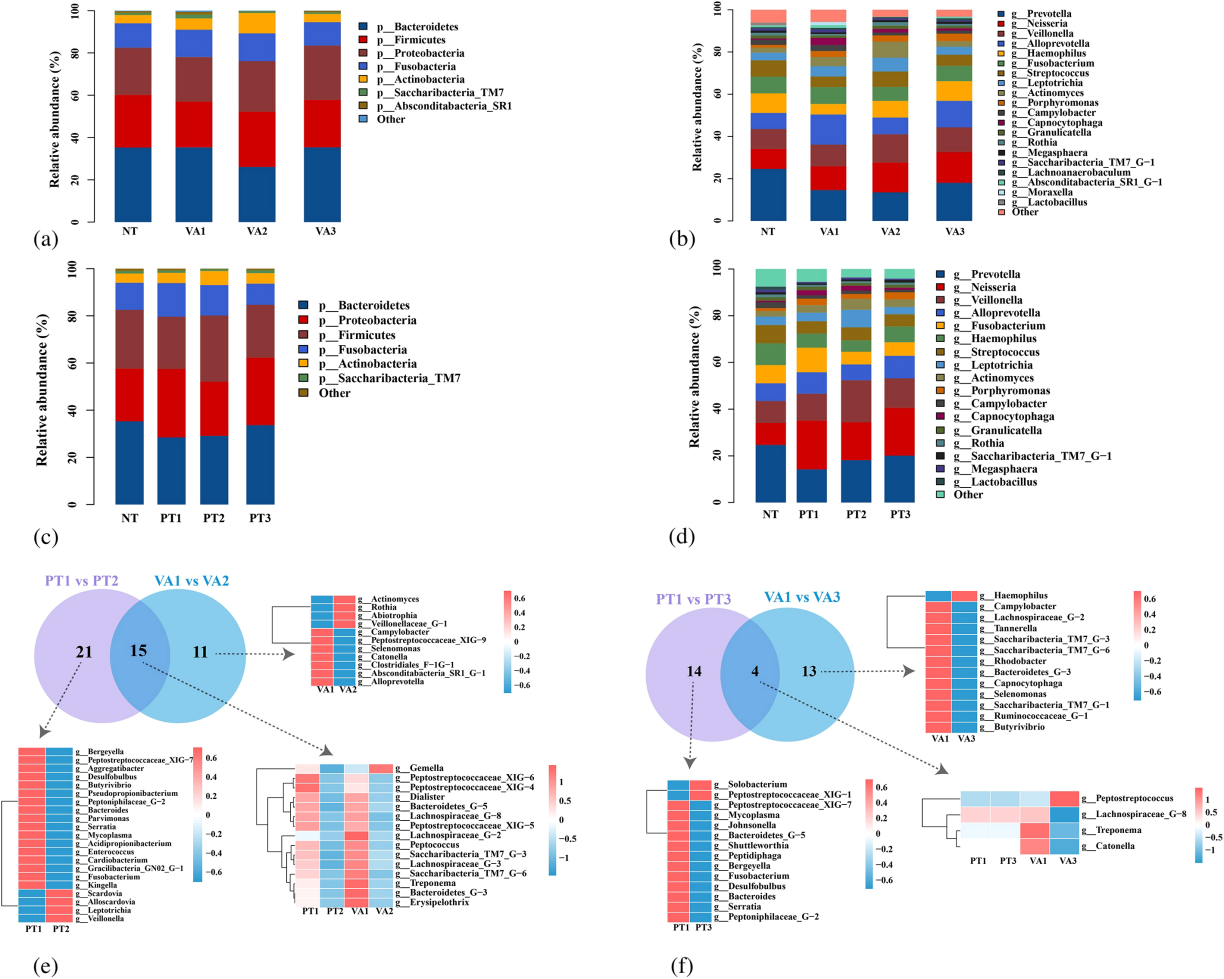
Comparison of tongue coating microbiota composition and structure between VA and PT groups. **(a)** Phylum-level composition of tongue coating microbiota in the VA group; **(b)** Genus-level composition of tongue coating microbiota in the VA group; **(c)** Phylum-level composition of tongue coating microbiota in the PT group; **(d)** Genus-level composition of tongue coating microbiota in the PT group; **(e)** Differences in bacterial genera between VA and PT groups at baseline vs. post-treatment; **(f)** Differences in bacterial genera between VA and PT groups at baseline vs. follow-up.

In the VA group, the top five dominant bacterial phyla at W0, W2, and W6 were *Actinobacteria*, *Fusobacteria*, *Proteobacteria*, *Firmicutes*, and *Bacteroidetes* (**Figure 6A**). Among these, *Actinobacteria* significantly increased at W2, while *Bacteroidetes* markedly decreased at the same time point. Both phyla returned to baseline levels at W6.

##### 3.4.3.2 Probiotics partially mitigated the disruption of the microbiota induced by VA regimen

The top five dominant bacterial phyla in the PT group at W0, W2, and W6 were identical to those observed in the VA group, namely *Actinobacteria*, *Fusobacteria*, *Proteobacteria*, *Firmicutes*, and *Bacteroidetes* (**Figure 6C**). The relative abundances of these top five dominant bacterial phyla in the PT group exhibited no significant changes before and after treatment, suggesting that probiotics partially mitigated the disruption of the microbiota induced by the 14-day VA regimen at the phylum level.

##### 3.4.3.3 Bacterial genus changes during treatment and follow-up in VA and PT groups

At W0, both VA and PT groups had the same top five dominant bacterial genera: Fusobacterium, AlloPrevotella, Veillonella, Neisseria, Prevotella. (**Figures 6B, D**) In the VA group, the top five shifted to AlloPrevotella, Veillonella, Neisseria, Prevotella, Hemophilus at W2 and remained consistent at W6. (**Figure 6B**) For the PT group, the top five at W2 were AlloPrevotella, Veillonella, Neisseria, Prevotella, Leptotrichia, and shifted to AlloPrevotella, Veillonella, Neisseria, Prevotella, Hemophilus at W6. (**Figure 6D**).

Comparisons of the relative abundances of the top five bacterial genera between the VA and PT groups were conducted across three time points: W0, W2 and W6. In the VA group, AlloPrevotella decreased significantly at W2 but returned to baseline at W6. Conversely, Hemophilus exhibited a continuous increase at W2, with its abundance at W6 being significantly higher than at baseline. In the PT group, Veillonella and Leptotrichia increased significantly at W2 but reverted to baseline at W6; the relative abundance of Clostridium was lower at both W2 and W6 compared to W0.

**Figure 6E** demonstrates the alterations in the levels of specific bacterial genera in both the VA and PT groups at W2 relative to W0. In the VA group alone, eleven bacterial genera exhibited FDR-adjusted significant changes in their relative abundance: seven genera showed a decrease, while four genera displayed an increase. Conversely, the PT group had twenty-one bacterial genera with FDR-adjusted significant changes in relative abundance, where seventeen genera decreased and four genera increased. Fifteen bacterial genera exhibited FDR-adjusted significant changes in relative abundance in both the VA and PT groups. Of these, 14 genera showed decreased relative abundance in both groups. In contrast, Gemella displayed decreased relative abundance in the PT group but increased relative abundance in the VA group.

**Figure 6f** demonstrates the alterations in specific bacterial genus levels in the VA and PT groups at W6 relative to W0. Thirteen bacterial genera exhibited FDR-adjusted significant changes in relative abundance exclusively in the VA group: 12 genera showed decreased relative abundance, whereas one genus (Haemophilus) displayed an increase. Fourteen bacterial genera displayed FDR-adjusted significant changes in relative abundance exclusively in the PT group, with 12 genera exhibiting decreased relative abundance and two additional genera showing increased relative abundance.

Four bacterial genera exhibited FDR-adjusted significant changes in relative abundance in both the VA and PT groups. Specifically, Peptostreptococcus exhibited increased relative abundance in the VA group, whereas Lachnospiraceae_G-8, Treponema, and Catonella showed decreased relative abundance in this group. While these four genera also displayed changes in the PT group, the magnitude of these alterations was not statistically significant after FDR correction.

#### 3.4.4 Probiotics increased the activity of carbohydrate metabolic pathways

**Figure 7:**
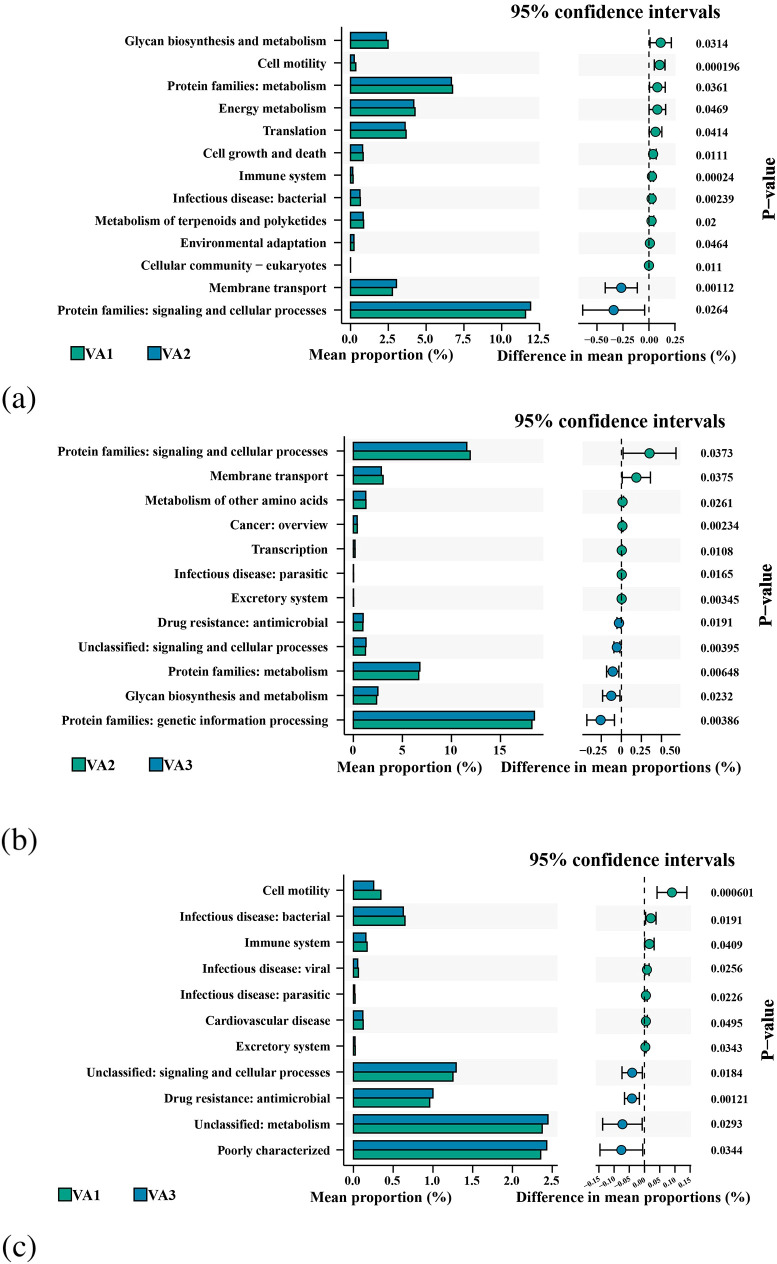
KEGG pathway enrichment analysis of VA group during *H. pylori* eradication. **(a)** Baseline vs. post-treatment; **(b)** Post-treatment vs. follow-up; **(c)** Baseline vs. follow-up.

**Figure 8:**
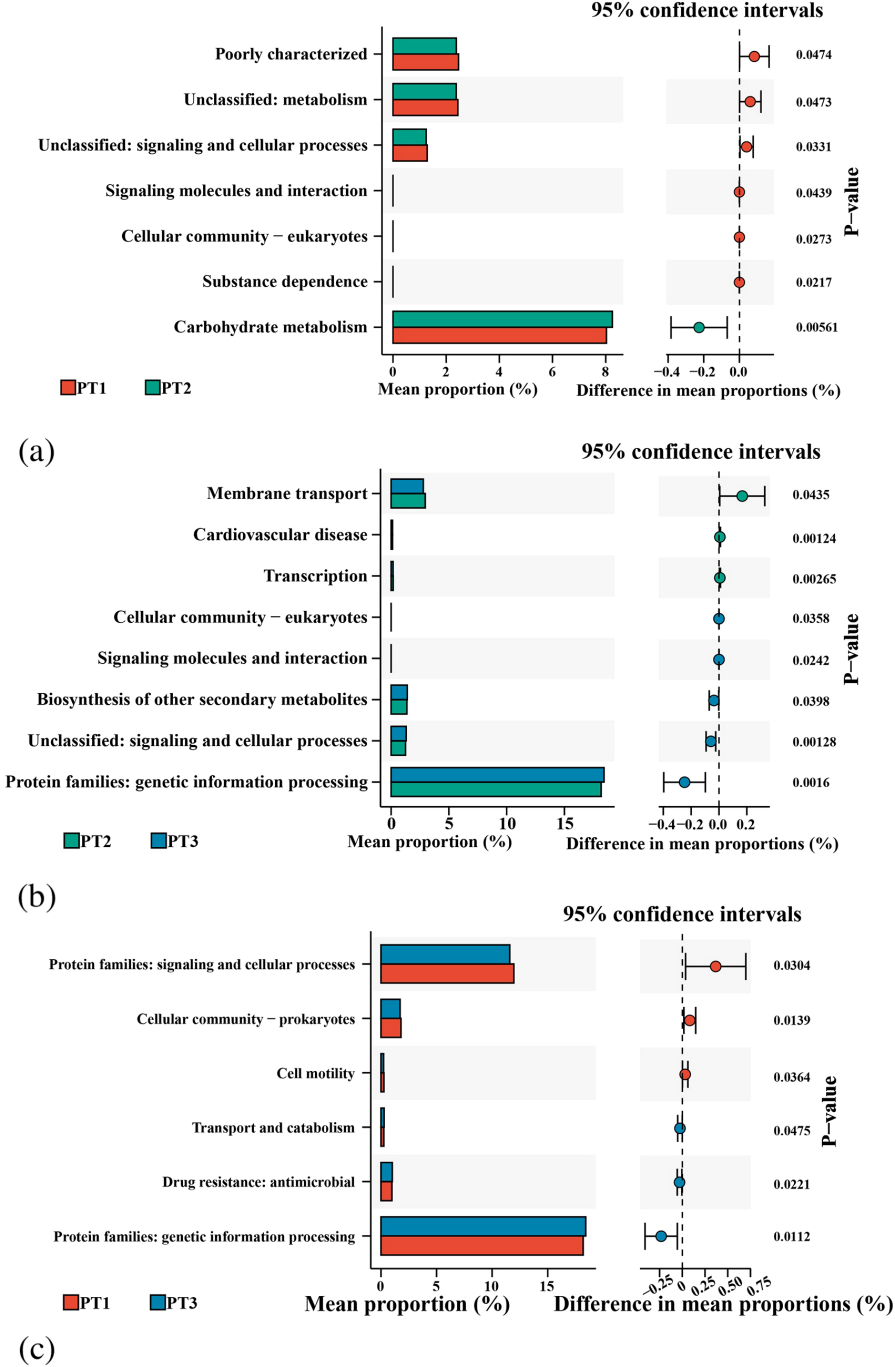
KEGG pathway enrichment analysis of PT group during *H. pylori* eradication. **(a)** Baseline vs. post-treatment; **(b)** Post-treatment vs. follow-up; **(c)** Baseline vs. follow-up.

Predicted KEGG pathway analysis based on PICRUSt2 revealed that eradication therapy for *H. pylori* led to FDR-adjusted significant changes in predicted pathway enrichment levels between different time points in the two groups. The perturbation of signaling pathways in the VA group was more pronounced than that in the PT group, with 13, 12, and 11 pathways showing distinct changes between VA1 and VA2 (**Figure 7A**), VA2 and VA3 (**Figure 7B**), and VA1 and VA3 (**Figure 7C**), respectively. In the probiotic group, there were 7, 8, and 6 pathways with FDR-adjusted significant differences between PT1 and PT2 (**Figure 8A**), PT2 and PT3 (**Figure 8B**), and PT1 and PT3 (**Figure 8c**), respectively.

After treatment in the VA group, pathways associated with “Cellular community-eukaryotes,” “Cell motility,” “Translation,” “Cell growth and death,” “Immune system,” “Infectious disease: bacterial,” “Environmental adaptation,” and metabolism showed reduced enrichment levels. In contrast, pathways such as “Membrane transport” and “Protein families: signaling and cellular processes” exhibited increased enrichment. Following eradication, the PT group showed decreased expression of pathways including “Cellular community-eukaryotes,” “Signaling molecules and interaction,” “Substance dependence,” and unclassified categories (“Unclassified: signaling and cellular processes; Unclassified: metabolism”). Conversely, the expression of the “Carbohydrate metabolism” pathway was upregulated.

## 4 Discussion

### 4.1 Effect of *H. pylori* infection on oral microbiota

The oral microbiota is important in the human micro-ecosystem and related to health (Peng et al., 2022). Numerous previous studies have demonstrated a certain association between *H. pylori* infection and the structure and diversity of the oral microbiota. Nevertheless, the relevant findings remain inconsistent due to factors including sample sources, personal habits, regional variations, and the number of participants. Some studies have demonstrated that *H. pylori* infection modifies the beta diversity of the oral microbiota while leaving alpha diversity unaffected (Liang et al., 2021). Conversely, another investigation reports that *H. pylori* colonization impacts both alpha and beta diversity of the oral microbial community (Ji et al., 2020). The results of the presented study demonstrated that both the Chao1 and Shannon indices in the Hp group were lower than those in the NT group, with significant differences also observed in β diversity between the two groups. The top five dominant bacterial phyla in both groups were Actinobacteria, Clostridium, Proteobacteria, Firmicutes, and Bacteroidetes, with no significant difference in relative abundance. No significant difference was observed in the relative abundance of these dominant phyla between the two groups, which aligns with previous research findings (Ji et al., 2020). Additionally, the relative abundances of Hemophilus and Prevotella were lower in the Hp group. These results indicate that *H. pylori* infection changes the composition of the oral microbiota in patients.

LEfSe analysis showed the Hp group had lower Lactobacillus and higher Moraxella relative abundance than the NT group. Notably, Lactobacillus has been reported to antagonize the pathogenicity of Porphyromonas gingivalis and contribute to the maintenance of oral health (Zhao et al., 2012; Zhao et al., 2019). Oral *Porphyromonas gingivalis* is implicated in the pathogenesis of periodontitis and oral cancer, while also being associated with cardiovascular disease and Alzheimer’s disease (Etta et al., 2023; Fu et al., 2023). Conversely, *Moraxella*, a well-recognized opportunistic pathogen, is linked to otitis media, keratitis, respiratory infections, and acute exacerbations of chronic obstructive pulmonary disease (Conway et al., 2023; Ding et al., 2024; Yagyu et al., 2025). These findings suggest *H. pylori* infection may impact oral and systemic health by reducing beneficial bacteria and increasing pathogenic taxa.

KEGG analysis revealed that the enrichment level of carbohydrate metabolism pathways in the oral microbiota genes of the Hp group was lower than that in the NT group, indicating that *H. pylori* infection impairs the carbohydrate metabolic capacity of the oral microbiota. Notably, impaired carbohydrate metabolic capacity of oral microorganisms has been reported to be associated with periodontitis (Belstrom et al., 2021). Additionally, studies have shown that subjects with *H. pylori* infection have a higher risk of periodontitis than non-infected individuals, and eradication therapy can reduce this risk (Adachi et al., 2019). Despite observed significant oral microbiota differences between *H. pylori*-infected patients and controls, the control group had a non-significant younger age trend. Sensitivity analyses adjusting for demographics did not alter core findings, but future strictly age-matched studies are warranted to exclude age-related confounding.

By integrating taxonomic and functional data, we propose a cascade model of *H. pylori*-induced oral microecological disruption: H. pylori infection first drives genus-level shifts (Lactobacillus reduction and Moraxella overgrowth), which impairs carbohydrate metabolic capacity, reduces short-chain fatty acid production, and ultimately compromises mucosal barrier and immune function, promoting further dysbiosis. This model clarifies the mechanism linking H. pylori infection to higher oral/systemic disease risk, emphasizing the crosstalk between microbial taxonomy and function in oral homeostasis maintenance.

### 4.2 Effect of *H. pylori* eradication on oral microbiota

This study compared the tongue coating microbiota of patients in the VA group at W0, W2, and W6, and found that the Chao1 index and Shannon index of the microbiota at W2 were both decreased compared to W0, but the change in the Shannon index was not statistically significant. PCoA indicated significant changes in the β diversity of the microbiota. All these changes recovered to baseline levels at W6. Previous relevant studies have confirmed that *H. pylori* eradication therapy can cause perturbation of the oral microbiota, and the degree of microbiota changes shows a positive correlation with the number of medications used. Ji et al. used a 14-day bismuth-containing quadruple therapy to eradicate *H. pylori*, and the analysis results showed that eradication therapy significantly reduced the α diversity of salivary bacteria and also significantly altered the β diversity of the microbiota (Ji et al., 2020). Suzuki et al (Suzuki et al., 2020). noted decreased oral microbiota diversity and community composition changes post-7-day triple therapy. Hu et al. observed no significant α/β diversity changes with 1-week/10-day VA dual therapy, differing from this study—likely due to increased amoxicillin dosage and prolonged treatment course in the current study (Hu et al., 2022).

Two weeks after *H. pylori* eradication therapy, significant changes were observed in the relative abundances of dominant bacterial phyla: Actinobacteria increased while Bacteroidetes decreased. Among dominant genera, Alloprevotella significantly declined at W2 but recovered to W0 levels by W6; Hemophilus abundance at W6 was significantly higher than baseline. These findings indicate the 14-day VA regimen perturbs oral microbiota at both phylum and genus levels, with most alterations reversing within a short period.

### 4.3 The impact of VA therapy combined with probiotics on eradication rate, adverse reactions, and oral microbiota

The Maastricht VI/Florence Consensus Report explicitly recommends appropriate probiotic supplementation in *H. pylori* eradication regimens. However, research on whether probiotics influence the 14-day VA therapy remains limited. In our study, PT and VA groups showed no statistical differences in eradication rates or adverse reaction incidences.

Current perspectives on probiotics’ precise role in oral microecology remain divided. Studies indicate probiotics can inhibit oral pathogens, disrupt pathogenic biofilms, and maintain microecological homeostasis (Nguyen et al., 2021; Radaic et al., 2022). Conversely, Liu et al. reported that probiotics had no significant effect on periodontal pathogens, while Dassi et al. observed higher α-diversity but unchanged β-diversity in oral microbiota of probiotic recipients (Dassi et al., 2018; Liu et al., 2022).

At present, few studies have explored probiotic co-administration during *H. pylori* eradication: He et al. recorded oral microbiota changes with probiotic-supplemented bismuth quadruple therapy, finding no intergroup difference in α-diversity pre/post-eradication, but significant β-diversity alterations in the placebo group (He et al., 2022). In our research, PT group’s Chao1 index decreased rapidly at W2 and recovered by W6, consistent with the VA group. While Shannon indices showed no statistical differences between groups pre/post-eradication, the PT group exhibited smaller fluctuations. Additionally, PT group’s β-diversity remained unaltered, and no significant changes were observed in the relative abundances of the top five dominant phyla. Among key genera, Fusobacterium decreased at W2, while Veillonella and Leptotrichia increased but recovered to baseline by W6.

These results suggest probioticspartially mitigate the oral microbiota perturbation caused by the 14-day VA regimen, contributing to relative oral microecological stability during therapy. However, it should be noted that even with probiotic supplementation, the oral microbiota composition remained distinct from that of *H. pylori*-negative controls at both post-treatment and follow-up time points, indicating that full restoration of microbial balance was not achieved within the 6-week follow-up period.

Notably, the negative control group exhibited significantly higher Chao1 and Shannon indices than both the PT and VA groups at W0, W2, and W6. PCoA analysis suggests that the β diversity of the negative control group is also different from the corresponding indicators detected in the PT and VA groups at W0, W2 and W6. These findings indicate that short-term oral microbiota alterations are associated with eradication therapy; while probiotics partially mitigate drug-induced perturbations to the oral microecology, restoring it to pre-infection levels remains challenging in the short term.

Probiotic supplementation further inhibited the colonization of oral pathogens including Fusobacterium, Mycoplasma and Serratia during eradication therapy. KEGG analysis indicated that probiotics partially mitigated VA regimen-induced impairment of microbial metabolic function, and specifically enhanced carbohydrate metabolism activity, which may reduce *H. pylori*-associated periodontitis risk and benefit oral health. Given that PICRUSt2 only provides predictive functional insights, these preliminary findings require further validation via metagenomic sequencing or targeted metabolomic analyses.

Although clinical oral health indices were not systematically collected in this study, the observed reductions in pathogenic genera including *Fusobacterium, Mycoplasma* and *Serratia*in the probiotic group align with established periodontal pathogen profiles. These microbial changes suggest potential clinical benefits that warrant further investigation in future studies with standardized oral health assessments.

In summary, this study found that Bacillus subtilis-Enterococcus faecium probiotic co-administration partially mitigates VA therapy-induced oral microbiota disturbances during his study found that Bacillus subtilis-Enterococcus faecium probiotic co-administration mitigates VA therapy-induced oral microbiota disturbances during *H. pylori* eradication, and innovatively revealed its multi-level (phylum/genus/function) synergistic protective mechanism for maintaining microecological stability, providing new insights into microbial structure-function associations. Limitations include small single-center sample, missing baseline confounding data (oral hygiene, diet, dental history etc.), unvalidated 16S-based functional predictions, and only 6-week short-term follow-up. Future multicenter studies with confounder adjustment, experimental validation and long-term follow-up are warranted to confirm the intervention effects. Limitations include small single-center sample, unvalidated functional predictions, short 6-week follow-up, and missing key confounder data. Future studies will adopt multicenter large-sample designs, validate functional findings via experiments, and extend follow-up to assess long-term effects.

In summary, this study found that Bacillus subtilis-Enterococcus faecium probiotic co-administration partially mitigates VA therapy-induced oral microbiota disturbances during his study found that Bacillus subtilis-Enterococcus faecium probiotic co-administration mitigates VA therapy-induced oral microbiota disturbances during *H. pylori* eradication, and innovatively revealed its multi-level (phylum/genus/function) synergistic protective mechanism for maintaining microecological stability, providing new insights into microbial structure-function associations. These exploratory findings suggest a potential protective effect of probiotics, but further experimental validation is required to confirm causal relationships and functional consequences of the observed taxonomic shifts. Limitations include small single-center sample, missing baseline confounding data (oral hygiene, diet, dental history etc.), unvalidated 16S-based functional predictions, and only 6-week short-term follow-up. Future multicenter studies with confounder adjustment, experimental validation and long-term follow-up are warranted to confirm the intervention effects. Limitations include small single-center sample, missing baseline confounding data (oral hygiene, diet, dental history etc.), unvalidated 16S-based functional predictions, and only 6-week short-term follow-up. Future multicenter studies with confounder adjustment, experimental validation and long-term follow-up are warranted to confirm the intervention effects. Limitations include small single-center sample, unvalidated functional predictions, short 6-week follow-up, and missing key confounder data. Future studies will adopt multicenter large-sample designs, validate functional findings via experiments, and extend follow-up to assess long-term effects.

In summary, this study found that Bacillus subtilis-Enterococcus faecium probiotic co-administration partially mitigates VA therapy-induced oral microbiota disturbances during his study found that Bacillus subtilis-Enterococcus faecium probiotic co-administration mitigates VA therapy-induced oral microbiota disturbances during *H. pylori* eradication, and innovatively revealed its multi-level (phylum/genus/function) synergistic protective mechanism for maintaining microecological stability, providing new insights into microbial structure-function associations. These exploratory findings suggest a potential protective effect of probiotics, but further experimental validation is required to confirm causal relationships and functional consequences of the observed taxonomic shifts. Limitations include small single-center sample, missing baseline confounding data (oral hygiene, diet, dental history etc.), unvalidated 16S-based functional predictions, and only 6-week short-term follow-up. Future multicenter studies with confounder adjustment, experimental validation and long-term follow-up are warranted to confirm the intervention effects. Limitations include small single-center sample, missing baseline confounding data (oral hygiene, diet, dental history etc.), unvalidated 16S-based functional predictions, and only 6-week short-term follow-up. Future multicenter studies with confounder adjustment, experimental validation and long-term follow-up are warranted to confirm the intervention effects. This study should be considered exploratory in nature, and definitive conclusions regarding clinical utility require confirmation in larger multicenter trials. The 6-week follow-up period was selected to align with standard clinical H. pylori eradication assessment protocols, but we acknowledge that this duration is insufficient to evaluate long-term microbiota stability or complete recovery to pre-infection states. Important confounding factors including oral hygiene practices, diet, dental history, and recent oral treatments were not systematically collected in this study, which may introduce unmeasured confounding. Future studies should include standardized assessment of these variables to improve the robustness of findings. Future studies will adopt multicenter large-sample designs, validate functional findings via experiments, and extend follow-up to assess long-term effects.

## Data Availability

The original contributions presented in the study are included in the article/supplementary material. Further inquiries can be directed to the corresponding author/s.
